# Tuning the Luminescence Response of an Air-Hole Photonic Crystal Slab Using Etching Depth Variation

**DOI:** 10.3390/nano13101678

**Published:** 2023-05-19

**Authors:** Artem V. Peretokin, Dmitry V. Yurasov, Margarita V. Stepikhova, Mikhail V. Shaleev, Artem N. Yablonskiy, Dmitry V. Shengurov, Sergey A. Dyakov, Ekaterina E. Rodyakina, Zhanna V. Smagina, Alexey V. Novikov

**Affiliations:** 1 Institute for Physics of Microstructures of the Russian Academy of Sciences, 603950 Nizhny Novgorod, Russia; aperetokin@ipmras.ru (A.V.P.); inquisitor@ipmras.ru (D.V.Y.); mst@ipmras.ru (M.V.S.); shaleev@ipmras.ru (M.V.S.); yablonsk@ipmras.ru (A.N.Y.); shen@ipmras.ru (D.V.S.); 2 Skolkovo Institute of Science and Technology, 143026 Moscow, Russia; s.dyakov@skoltech.ru; 3 Rzhanov Institute of Semiconductor Physics, Siberian Branch of Russian Academy of Sciences, 630090 Novosibirsk, Russia; rodyakina@isp.nsc.ru (E.E.R.); smagina@isp.nsc.ru (Z.V.S.); 4 Physical Department, Novosibirsk State University, 630090 Novosibirsk, Russia; 5 Radiophysical Department, Lobachevsky State University of Nizhny Novgorod, 603950 Nizhny Novgorod, Russia

**Keywords:** Ge self-assembled islands, photonic crystal slab, etching depth, micro-photoluminescence, photonic crystal modes, bound states in continuum

## Abstract

Detailed studies of the luminescent properties of the Si-based 2D photonic crystal (PhC) slabs with air holes of various depths are reported. Ge self-assembled quantum dots served as an internal light source. It was obtained that changing the air hole depth is a powerful tool which allows tuning of the optical properties of the PhC. It was shown that increasing the depth of the holes in the PhC has complex influences on its overall photoluminescence (PL) response due to the simultaneous influences of counteracting factors. As a result, the maximal increase in the PL signal of more than two orders of magnitude was obtained for some intermediate, but not full, depth of the PhC’s air holes. It was demonstrated that it is possible to engineer the PhC band structure in such a way as to construct specific states, namely bound states in continuum (BIC), with specially designed dispersion curves being relatively flat. In this case, such states manifest themselves as sharp peaks in the PL spectra, and have high Q-factors which are larger than those of radiative modes and other BIC modes without such a flat dispersion characteristic.

## 1. Introduction

Nowadays, the explosive growth of the volume of generated and transmitted information requires ever-increasing data transfer rates. It is widely accepted that the development of photonic components integrated with modern semiconductor microelectronics would be the mainstream direction for this purpose. Given that electronics are based on silicon, the photonic blocks also have to be Si-compatible. Despite the remarkable progress in the development of various integrated photonic components, such as modulators, multiplexers, and detectors, the fabrication of a compact, efficient, and Si-friendly near-infrared light source is still a challenge. Although the most successful approach is based on the integration of internally efficient light sources, mostly based on III-V direct band semiconductors, with silicon, a number of difficulties still exist for this method [1,2,3,4]. From the integration point of view, an alternative approach using group-IV structures is far more attractive. Unfortunately, group-IV elements such as Si and Ge are indirect gap semiconductors and so they emit light very inefficiently. In view of this, two main directions were developed to pursue the enhancement of their light emission efficiency. The first direction was based on modification of the active medium band structure, attempting to make it direct or quasi-direct by using strained and/or heavily n-doped Ge [5,6], alloying Ge or SiGe with Sn [7,8], constructing hexagonal Si/Ge core-shell nanostructures [9], or defect engineering in structures with Ge self-assembled quantum dots (QDs) [10,11]. The second direction was devoted to the modification of the surroundings of an active medium i.e., to the formation of various microresonators. They include plasmonic [12,13] and dielectric resonators such as photonic crystals [14,15,16,17], Mie resonators [18,19,20,21,22,23,24,25], and metasurfaces [26,27,28,29]. To be fair, we note that the latter approach has proved to be powerful for various material systems. All-dielectric resonators have the significant advantage of very low ohmic losses as compared to metallic resonators, and so they are preferable for light manipulation. In a variety of dielectric microresonators, the photonic crystals (PhCs) were investigated most thoroughly, and a number of bright results have been presented to date. Nevertheless, there is still plenty of room in this field for, for example, the generation of novel complex designs with the help of neuromorphic computation [30,31,32] or for the exploitation of astonishing states such as bound states in continuum (BIC) or topological states which PhCs could support, which may provide delicate yet powerful control of light in PhCs [33,34,35,36,37].

In this article, we have investigated the air-hole two-dimensional PhC slabs fabricated on SOI-based structures with the embedded Ge QDs serving as internal light sources. On the one hand, such a kind of structure is relatively easy to fabricate and the process flow is compatible with common microelectronic fabrication technology. On the other hand, using Ge QDs as internal sources of near-infrared light with relatively wide emission spectrum allows to study the optical properties of the PhCs rather conveniently [38,39,40]. It should be noted that, among a large number of papers devoted to the air-hole type of PhCs, the majority of them considered only structures in which the air holes were etched through the whole structure, thus providing the maximal refractive index modulation. However, variation of the air hole depth could also be a tool for modification of the PhC’s optical properties. Only a few works have considered the variation of the holes’ depths in PhCs [41,42], but in these works mainly paid attention to the enhancement of the light extraction efficiency in the PhC surface emitting lasers, the impact of non-radiative surface recombination, and the spectral shift of a laser line. However the features of the PhC band structure and the peculiarities of different PhC modes have not been studied. The possibilities of the modification of the PhC mode structure, selection, and of the spectral tuning of some specific PhC modes have not been considered. Furthermore, when the active light emitting medium is embedded inside the structure (in our case, Ge QDs embedded in a Si matrix), the fabrication of holes will not only create a PhC, but will also remove some part of the active medium and will significantly enhance the surface-to-volume ratio of the structure. The latter will lead to the increase in surface recombination [14,15,38,43], which is detrimental in view of the overall emission efficiency of the structure. In this paper, we have fabricated PhCs with air holes of various depths and studied their optical properties. We have demonstrated that the hole depth in PhCs is a viable tool to engineer the mode structure of a PhC, and so to tune the optical response in such a kind of structure. It was also obtained that the maximal PL response is reached at some intermediate but not full depth of the air holes. The peculiar features observed in the PL response of these PhCs, namely the appearance of a stop band, the manifestation of the bound states in the continuum, and the bound states in the continuum with nearly zero group velocity, were analyzed both experimentally and theoretically.

## 2. Materials and Methods

**Figure 1:**
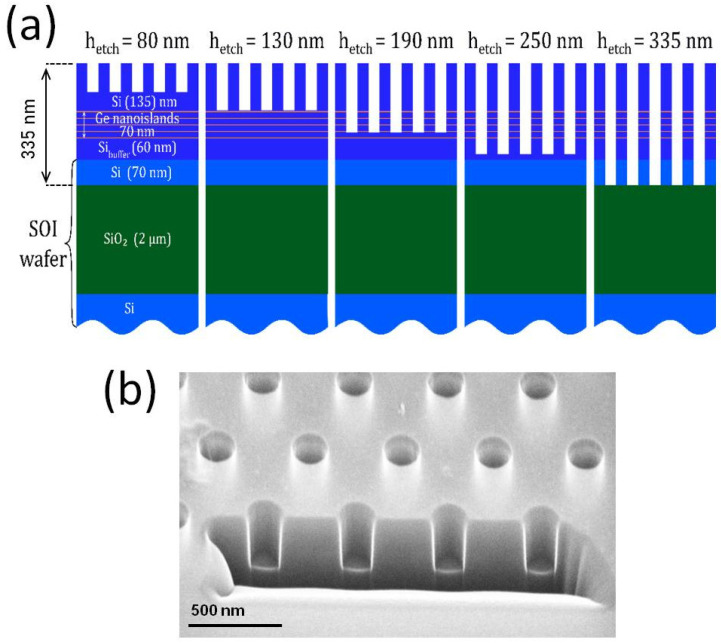
(**a**) Sample schematic for all 5 fabricated chips containing the PhCs with various hole depths. The layer thicknesses as well as hole’s depths are highlighted. (**b**) SEM image of the one of PhCs subjected to FIB milling.

Samples under investigation represented the air-hole photonic crystal (PhC) slabs with different PhC parameters and various air-hole depths (Figure 1). The initial structure was grown by molecular beam epitaxy on a SOI wafer having a 70 nm thick top Si layer and 2 µm thick buried oxide. The SOI wafer was ex situ cleaned using the modified RCA procedure with a final short dip in diluted HF acid and in situ annealed in a vacuum chamber which allowed to obtain an atomically flat Si surface before epitaxial growth. Firstly, a 60 nm thick Si buffer layer was deposited to move away from any possible remaining surface contaminations. Then five layers of Ge self-assembled QDs were formed via Stranski–Krastanow growth mode at 620 °C, which served as an active medium. The amount of deposited Ge was ~1 nm and Si separation layers between QDs were ~17 nm thick. Capping the Si layer at 135 nm thick finalized the growth in such a way as to place the active medium in the center of the (future) slab. Total slab thickness was 335 nm as measured by scanning electron microscopy (SEM) and ellipsometry. After growth, the sample was divided into 5 chips. Electron beam lithography, followed by inductively coupled plasma reactive ion etching (ICP RIE), was used to fabricate the PhCs with a hexagonal structure and various parameters (hole periods and radii). The PhC period *a* (i.e., the hole spacing) varied in the range of *a* = 500–700 nm, while the hole radius *r* varied in such a way as to maintain an *r*/*a* ratio equal to *r*/*a* = 0.25. The PhC designs were the same for all 5 chips. Additionally, for each chip, an additional control sample was created, which represented nearly the same structure with several relatively large PhCs. The working chips and corresponding control samples underwent the ICP RIE process simultaneously. The etching times during the ICP RIE process were different for each pair of working and control samples, which allowed to obtain PhCs with the same geometrical parameters but with air holes of various depths (Figure 1). The satellites were further cleaved and the depths of etched air holes were determined by the side-view SEM. Additionally, for a few PhCs on the working chips, focused ion beam (FIB) milling was used in order to get access to the depths of the air holes independently. The results were nearly the same for both of the two methods, and the obtained depths of the holes are indicated in Figure 1a.

**Figure 2:**
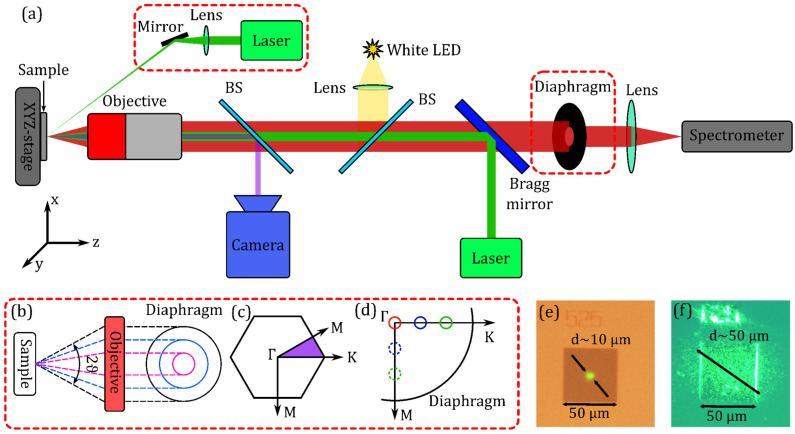
(**a**) Micro-PL schematic. Two red-dashed rectangular blocks represent additional elements which were used to study the PhC band structure. A white LED and camera were used for visualization and focusing the excitation laser spot in the PhC area. BS—beam splitter. (**b**) Illustration of PL collection angle variation by changing the diaphragm hole diameter. Different colors indicate different collecting angles. (**c**) First Brillouin zone and irreducible representation marked in violet. (**d**) Schematically illustrated scanning along Γ-K and Γ-M directions. Different colors indicate different measured points in reciprocal space. (**e**) Camera snapshot of the PhC and green pumping spot in standard micro-PL measurement. (**f**) Camera snapshot of the same using side-pumping.

The optical properties of the fabricated samples were studied using micro-photoluminescence (micro-PL) spectroscopy at room temperature (see Figure 2). Optical excitation was carried out using the laser module (*λ_exc_* = 532 nm) and the «Mitutoyo» M Plan APONIR 10× objective (NA = 0.26). The excitation spot size was ~10 µm. The PL was collected using the same objective and detected by the high resolution Fourier spectrometer Bruker IFS 125 HR with a cooled Ge detector. In some experiments, the standard micro-PL technique was modified, and two additional red-dashed rectangular blocks in Figure 2 represent these modifications. These blocks were introduced to measure the luminescent response of PhCs in the vicinity of the Γ-point of the Brillouin zone with different collection angles (i.e., within the different ranges of wave vectors in reciprocal space), as well as to measure the PhC band structure. The PL collection angle was changed with the help of a set of diaphragms with various diameters (Figure 2b) placed in the parallel beam formed by an objective. The measurements with the diaphragm in the center of the parallel beam corresponded to the measurements in the vicinity of Γ-point of the Brillouin zone. Shifting such a diaphragm in the XY-plane towards the Γ-K and Γ-M directions of a PhC’s Brillouin zone (see Figure 2c,d) allowed us to scan the band structure of the PhCs experimentally. In such a kind of measurement, we used the side-excitation schema (top red-dashed block in Figure 2a) instead of the standard one using the Bragg mirror, which allowed to enlarge the excitation spot up to ~50 µm (Figure 2f). The large spot provided the excitation of nearly the whole PhC while maintaining the same excitation power density. This increased the collected signal and enabled us to work with small diaphragms, and therefore with a higher resolution in *k*-space.

Theoretical modeling of the PhC band structure and the mode’s emissivity was performed using rigorous coupled wave analysis [44].

## 3. Results

### 3.1. Theoretical Background

**Figure 3:**
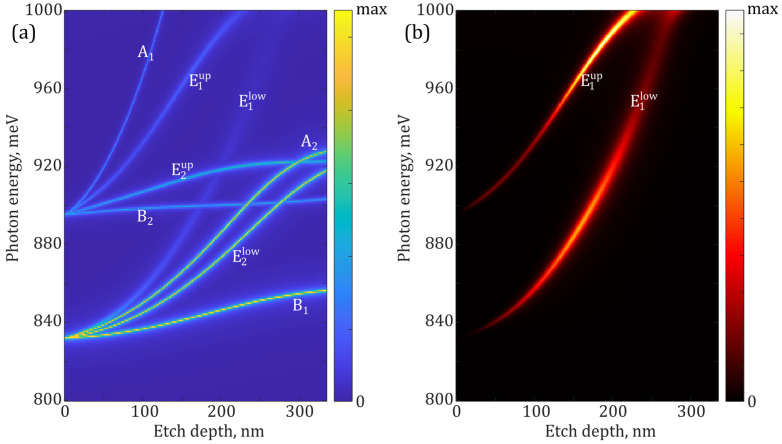
(**a**) Norm of a scattering matrix in the Γ-point calculated for a PhC with a hexagonal structure with *a* = 550 nm, *r*/*a* = 0.25 for various etching depths. The right edge of the plot corresponds to the hole depth of 335 nm, coinciding with total slab thickness. (**b**) Emissivity in the Γ-point for modes plotted in Figure 3a. Only two leaky modes remain while BIC modes vanish.

The infinite planar Si/SiO_2_ waveguide without any holes supports waveguide modes, which are converted into PhC modes when the air holes are introduced (i.e., when the pattern of the periodic refractive index is created). Moving from “weak” grating where the modulation of the refractive index is small to “strong” grating, one could observe the modes splitting, their energy shifting and bending [45,46]. Moreover, the energy shift of different modes can be different, which leads to the modes’ convergence, thereby increasing the likelihood of their interaction. To illustrate this, we present in Figure 3 the results of theoretical simulations carried out for modes supported by PhCs realized in the experiment. The modes’ energies and their emissivity depending on the hole’s depths (modulation of the refractive index) are shown. PhCs with hexagonal lattices studied in this work support 12 fundamental modes: two of them are the radiation (leaky) doublets: $E_{1}^{low}$ and $E_{1}^{up}$, another two—BIC doublets: $E_{2}^{low}$ and $E_{2}^{up}$, and four of them are BIC singlets: $A_{1}$, $A_{2}$, $B_{1}$, and $B_{2}$ [46,47]. The theoretical results clearly show the spectral behavior and convergence of some of PhC modes with the increasing depth of the holes, and reveal the specific behavior of BICs. The BIC modes are characterized by the vanishing emissivity (and infinite Q-factors) in the Γ-point of the PhC’s Brillouin zone. This is clearly seen from the comparison of Figure 3a,b where the emissivity of various modes in the Γ-point is plotted. The emissivity of different modes was simulated for the ideal case of a laterally infinite PhC slab. One can see in Figure 3b that only two radiative modes ($E_{1}^{low}$ and $E_{1}^{up}$) have non-vanishing emissivity, whereas the BIC modes in the ideal PhC are completely dark. On the other hand, the leaky modes would have moderate Q-factors. Here we should mention that BIC states could exist only in the infinite structures and any limitations such as finite thickness, fabrication imperfections, deflection from the Γ-point, etc. transform them into quasi-BIC states, which are no longer non-emitting, but still may have rather large Q-factors [34] and references therein.

**Figure 4:**
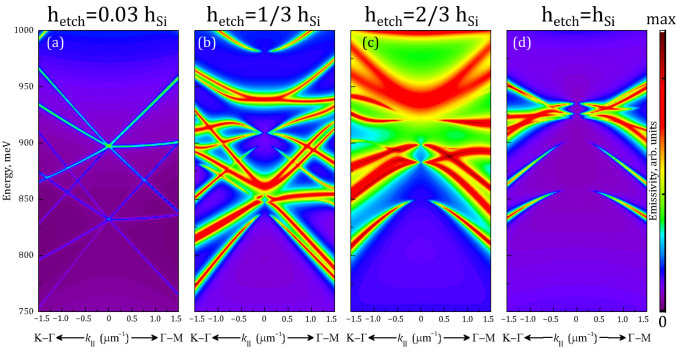
Far field emissivities of the PhC modes calculated near the Γ-point for the different etching depths (*h_etch_*). *h_etch_* increases in (**a**–**d**) as indicated above the corresponding images. *h_Si_* is the total slab thickness, being equal to 335 nm. The PhC geometrical parameters were *a* = 550 nm and *r*/*a* = 0.25. Emissivity is in log scale, and is common for all 4 panels.

Detailed theoretical simulations of modes’ emissivity in the vicinity of the Γ-point of the Brillouin zone that were performed for PhCs with varied depth of air holes are shown in Figure 4. The parameters of the simulated PhCs were *a* = 550 nm and *r*/*a* = 0.25. One can see that for very shallow holes the modes of the PhCs just begin to form and their emissivity is rather low (Figure 4a). Increase in the depths of the holes causes the PhC modes to be far more pronounced and also leads to their energy shift. For the considered PhC parameters, when the holes’ depths are approximately 1/3 of the total slab thickness, the modes shift to higher energies and bend (Figure 4b), thus becoming spread over the relatively large spectral range. In this case, one may anticipate rather wide PL spectrum, especially for large PL collecting angles. When the holes become deeper (Figure 4c,d), the modes shift further in energy, but with different shifting rates for different modes. This results in some modes moving away from the spectral range of interest (750–1000 meV), but also in spectral convergence of certain modes, promoting their interaction. The modes of the same symmetry can efficiently interact, resulting in some kind of bending and repulsion, and even in interference, the consequence of which is called accidental BICs [34]. This may also lead to the flattening of their dispersion curves in some cases, and the narrowing of the covered spectral range. In this latter case, one can expect to observe the intense narrower PL lines, which are characterized by their extremely high quality (Q) factors (but the integrated PL intensity could be even smaller). Thus, increase in the depths of the holes will lead to the more resonant interaction of the active medium with the PhC modes, but etching holes deeper inside the structure will also lead to the removal of a certain part of the active medium and to an increase in surface non-radiative recombination on the developed sidewalls [14,38,48,49]. These factors have a detrimental impact on the overall light emission efficiency of the structure. Thus, an increase in depths of the holes will lead to the simultaneous action of multidirectional factors, and so may result in complex behavior of the structure’s PL response with varying etching depth.

### 3.2. Experimental Results and Discussion

**Figure 5:**
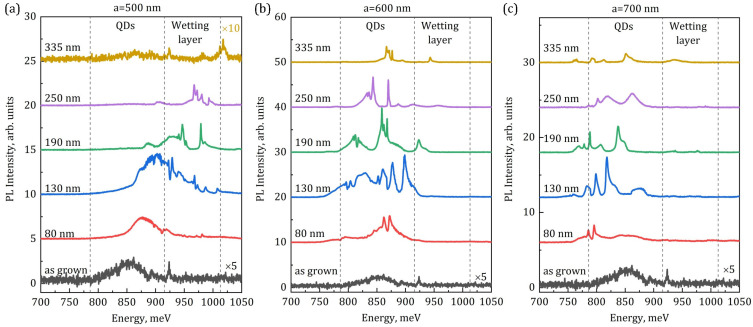
PL spectra for the as-grown sample and PhCs with *a* = 500 nm (**a**), *a* = 600 nm (**b**), and *a* = 700 nm (**c**). The ratio *r*/*a* = 0.25 is fixed for all PhCs. Etching depths are indicated near each spectrum. Vertical dashed lines denote the emission range related to the Ge QDs and to the wetting layer. Some spectra were multiplied for better visualization as indicated near corresponding curves.

The fabricated PhCs were examined by micro-PL spectroscopy at room temperature. Results for the PhCs with periods *a* = 500, 600, and 700 nm and *r*/*a* = 0.25 and various etching depths are summarized in Figure 5.

The initially grown sample shows a relatively broad PL signal centered around 850 meV, which is associated with Ge QDs (Figure 5, bottom curve), which is consistent with previous works [50,51,52]. The isolated, rather sharp peak at 925 meV is supposed to be defect-related, and possibly originated from some defects in the thin top Si layer of a SOI wafer. It is known that the wetting layer (WL) could also contribute to the PL signal in the energy range of 900–1000 meV [51], but at the experimental conditions used, the WL-related signal in the as-grown sample is rather weak and could not be reliably distinguished.

The PL response of PhCs shows a set of various lines corresponding to different PhC modes which depend on PhC parameters. An increase in the PhC period *a* leads to the mode’s shift to lower energies [53] (from part (a) to (c) in Figure 5). This allows to tune the PhC modes to WL- or QD-related signals (Figure 5a,b). For *a* = 600 nm, the PhC modes coincide well with the Ge QD emission range, which results in larger PL enhancement as compared to the PL of the as-grown sample (Figure 5b). Further increase in the PhC period to *a* = 700 nm leads to a partial decrease in the spectral matching and to a consequent decrease in the overall PL response (Figure 5c).

Change of the air-hole depth is another tool to modify the PhC’s band structure. The PhC PL response shifts to higher energies with the increase in the hole depth (Figure 5, it is mostly clear for *a* = 500 nm). Moreover, the PL shape also changes significantly with variation in hole depth. For the relatively small depth of 80 nm, the spectrum is relatively broad, but some narrow peaks could be distinguished. The overall PL enhancement in this case is probably mostly due to the increased light out-coupling. Further increase in the depth of the holes to 130 nm results in much more pronounced peaks in the PL spectra and further enhancement of the PL intensity. In this case, the active layer of Ge QDs is still not disturbed by etching and the non-radiative recombination on sidewalls is potentially not so large. The integrated PL enhancement is maximal among all cases (nearly 18 times). Further increase in the depths of the holes leads to the narrowing of the PL peaks, but the integrated PL intensity starts to decrease. Finally, for the maximal depth of the holes, the PL decreases significantly due to the increase in non-radiative surface recombination. The shortening of carrier lifetime in such a kind of structure, to below 1 ns values due to the surface recombination, was noticed previously [38,49], which underlines the impact of surface recombination. The PL decrease is, in part, also related to the poor localization of electrons in the Ge QD surrounding [54]—electrons could easily leave the small potential well in the vicinity of Ge QDs, and so could move in a lateral direction, thus enhancing the probability to recombine non-radiatively on the sidewalls. Another reason for the decrease in PL signal observed in PhCs with a full etching depth is the removal of the silicon layer beneath the Ge QDs, whose contribution to the generation of charge carriers and, accordingly, to the excitation of Ge QDs can be significant.

**Figure 6:**
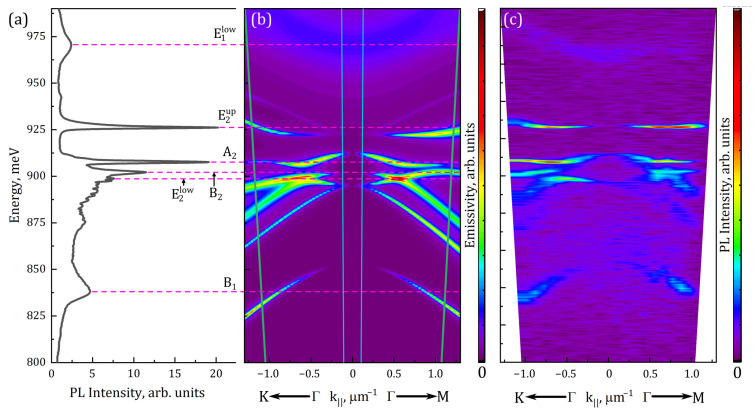
(**a**) Experimental PL spectrum measured in standard micro-PL setup for PhC with *a* = 550 nm, *r*/*a* = 0.25, and hole depth of 250 nm. Calculated (**b**) and measured (**c**) dispersion dependencies of modes’ emissivity for the same PhC (green and blue inclined lines in figure (**b**) correspond to the PL collecting angles of 30° and 3°, respectively). The panels are aligned by energy so one could directly compare the experimental peak positions and mode locations. Horizontal dashed lines are the guide for the eye for such a comparison.

Theoretical calculations presented above, in Figure 4, show that intermediate etching depths (i.e., smaller than the full slab thickness) may potentially be more interesting in some cases. We will further consider the PhC with *a* = 550 nm, *r*/*a* = 0.25, and a hole depth of 250 nm. The PL spectrum of this PhC is presented in Figure 6a along with its calculated mode structure, which is presented in Figure 6b. For this PhC, the narrowest PL lines among all studied samples were observed. It is also necessary to highlight the presence of a “stop band” in the band structure of this PhC. This is a “gap” in the energy range of 911–922 meV where no PhC modes exist for a broad range of *k*_‖_ values (Figure 6b). This band appears as a sharp dip in the PL spectrum (Figure 6a). Theoretical modeling revealed that the most intensive line in the PL spectrum corresponds to the BIC mode $E_{2}^{up}$. It is known that, ideally, the BIC mode would have an infinitely large Q-factor, and its emissivity disappears in the Γ-point, but all kinds of imperfections (in a wide sense) convert it to the mode which could emit light, but the Q-factor could remain rather high. In our case, the BIC mode $E_{2}^{up}$ also has another feature: its dispersion curve (for the lower branch) is relatively flat. This was directly observed by measuring the PL spectra (Figure 6c) for a set of different diaphragm shifts in lateral plane (along the symmetry directions of the PhC). This corresponds to the measurement at non-zero values of *k*_‖_ and so tracks the mode’s dispersion curves (as described in Section 2). The overall agreement between experimental and theoretical pictures is quite good. Some discrepancies may originate from uncertainties in the measurement of the PhC parameters and from some possible errors in the PL setup alignment and diaphragm positioning. A seeming discrepancy for the B_1_ mode is explained by its very low emissivity at small *k*_‖_ values [46] and the rather high slope of its dispersion curve. This mode is detected only when the *k*_‖_ values are large enough, and so the detected B_1_-related peak position shifts remarkably to lower energies. What is more interesting is that the shifts of the line corresponding to the $E_{2}^{up}$ mode are much smaller in energy as compared to other modes when the *k*_‖_ vector varies from 0 to 1 μm^−1^, thus confirming its flatter dispersion. The latter results in very low group velocity in the studied range of wave vectors *k*_‖_ (from −1 to +1 μm^−1^), as compared to other modes. As a result, this mode should have rather weak dependence on the PL collection angle and high Q factor.

**Figure 7:**
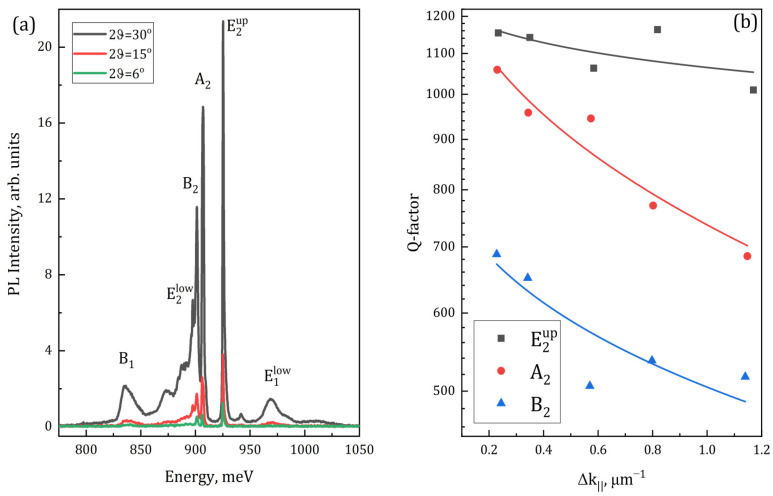
(**a**) PL spectra for the same PhC as presented in Figure 6, recorded using different collecting angles. These angles are indicated in the inset. (**b**) Dependences of measured Q-factors for different modes on the range of collected *k*_‖_.

In order to check on it and to obtain deeper insight into the mode’s behavior, the luminescent properties of this PhC were investigated using different PL collecting angles (Figure 7). This was carried out with the help of the set of diaphragms with different diameters (see Figure 2b). The increase in the PL collecting angle is equal to the increase in the range of photon wave vectors *k*_‖_ which could be detected. This results not only in the increase in the PL signal intensity, but also in the broadening of the spectrum, because in most cases the modes do not have a flat dispersion. In other words, the extension of the detected range of *k*_‖_ increases the energy range which is covered by a certain mode, resulting in the broadening of the corresponding peak in the PL spectrum. This is clearly seen for the $B_{1}$, $E_{2}^{low}$, $B_{2}$ and $E_{1}^{low}$ modes while for the mode $E_{2}^{up}$ such broadening is much smaller due to its flatter dispersion (Figure 6a,b). The latter is illustrated in Figure 7b, where the Q-factors are presented for some of the most intense modes: $E_{2}^{up}$,$\;A_{2}$, and $B_{2}$. Although all of them are the BIC modes, which should have much higher Q-factors as compared to the radiative mode $E_{1}$, their Q-factor dependencies on the collecting angle are not the same due to different dispersion curve slopes. Indeed, for the $E_{2}^{up}$ mode, the Q-factor is initially higher and decreases *slower* with the extension of the *k*_‖_ range that is collected. Thus, it hints at the following design rule: if one needs to achieve the narrow linewidth in such a kind of structure, the usage of “flat BIC” modes will be the favorable choice. It should be noted also that we have obtained sharper a PL line for the doublet ($E_{2}^{up}$) then for the singlet (*A*_2_), which seems to be somewhat counterintuitive. Nevertheless, it is fairly explained by the “flat” dispersion of the mode $E_{2}^{up}$—when the *k*_‖_ becomes large enough to distinguish two branches of the doublet $E_{2}^{up}$, they could be measured separately. The upper branch of this doublet mode shifts upwards in energy while the lower one nearly stands still. Thus, its linewidth could be reliably measured because it is not affected by the neighborhood of the upper branch of $E_{2}^{up}$ anymore. In the latter case, the advantage of “flat” dispersion of $E_{2}^{up}$ mode is manifested in the narrower PL line and weaker broadening with the increase in PL collection angle (Figure 7b).

The above mentioned features of the $E_{2}^{up}$ mode also manifested themselves experimentally by rather large enhancement of the peak PL intensity as compared to the as-grown sample, namely by 275 times. Such a high value is unlikely to be explained by only the enhancement of light extraction efficiency. The second well known reason for the PL enhancement—the Purcell effect—could also make a contribution. The direct way to estimate the Purcell effect is to measure the temporal PL dependences in comparison with the as-grown sample. However, in the Ge QDs/Si system at room temperature, the PL decay times are governed by the non-radiative recombination processes because type II band alignment results in relatively long radiative lifetimes [55,56]. So the possible shortening of the *radiative* lifetime due to the Purcell effect is masked by initially much shorter non-radiative lifetimes. As a result, the Purcell effect in this system is not so easy to assess experimentally, and it would be the subject of our future work.

## 4. Conclusions

In this work the PL response of Si-based PhC slabs with embedded Ge QDs was investigated for various depths of air holes which form a PhC. It was obtained that moderate hole depths are helpful to enhance the integrated PL response of Ge QDs. When the etched holes became, on the one hand, sufficiently large to form a PhC, but on the other hand still did not disturb the embedded active medium, the highest enhancement of the integrated PL intensity by ~18 times was obtained. Achievement of narrow PL lines requires larger hole depths at which the PhC modes are completely formed, and so the resonant interaction with such modes could be exploited. However, increasing the hole depth leads to more pronounced non-radiative recombination on the hole sidewalls, and also to the partial removal of the active medium. As a result of the simultaneous action of these counteracting factors, the optimal hole depth does not coincide with the full slab thickness. In our case, the maximal peak PL intensity enhancement by more than 2 orders of magnitude was observed for the hole depth of 250 nm (approximately ¾ of the full slab thickness), while further increase in the depth resulted in a decrease in the PL signal. A detailed study of the mode structure of PhCs revealed that it is possible to engineer the BIC modes with rather flat dispersion curves which could be used to get rather sharp PL lines. The Q-factors for such “flat BIC” modes are remarkably higher than for the other BIC modes without such a flat dispersion, and they show weaker dependence on the PL collection angle. Finally, it can be concluded that for the air-hole PhC slabs, the hole depth variation is a powerful tool for engineering the PL response from such structures.

## Data Availability

Not applicable.
